# Intra-Arterial Super-Selective Delivery of Yttrium-90 for the Treatment of Recurrent Glioblastoma: In Silico Proof of Concept with Feasibility and Safety Analysis

**DOI:** 10.3390/pharmaceutics17030345

**Published:** 2025-03-07

**Authors:** Giulia Paolani, Silvia Minosse, Silvia Strolin, Miriam Santoro, Noemi Pucci, Francesca Di Giuliano, Francesco Garaci, Letizia Oddo, Yosra Toumia, Eugenia Guida, Francesco Riccitelli, Giulia Perilli, Alessandra Vitaliti, Angelico Bedini, Susanna Dolci, Gaio Paradossi, Fabio Domenici, Valerio Da Ros, Lidia Strigari

**Affiliations:** 1 Department of Medical Physics, IRCCS Azienda Ospedaliero-Universitaria di Bologna, Via Massarenti 9, 40138 Bologna, Italy; giulia.paolani@ausl.re.it (G.P.); silvia.strolin@aosp.bo.it (S.S.); miriam.santoro@aosp.bo.it (M.S.); lidia.strigari@aosp.bo.it (L.S.); 2 U.O.C Diagnostic Imaging, Department of Integrated Care Processes, Fondazione PTV Policlinico “Tor Vergata”, University of Rome “Tor Vergata”, Viale Oxford 81, 00133 Rome, Italy; silvia.minosse2@gmail.com; 3 Department of Biomedicine and Prevention, University of Rome “Tor Vergata”, Viale Oxford 81, 00133 Rome, Italy; noemi.pucci@students.uniroma2.eu (N.P.); francesca.di.giuliano@uniroma2.it (F.D.G.); garaci@gmail.com (F.G.); eugenia.guida@uniroma2.it (E.G.); dolci@uniroma2.it (S.D.); darosvalerio@gmail.com (V.D.R.); 4 Department of Chemical Science and Technologies, University of Rome “Tor Vergata”, Via della Ricerca Scientifica 1, 00133 Rome, Italy; letizia.oddo@gmail.com (L.O.); yosra.toumia@roma2.infn.it (Y.T.); fra.riccitelli@gmail.com (F.R.); giulia.perilli@uniroma2.it (G.P.); alessandra.vitaliti@uniroma2.it (A.V.); paradossi@stc.uniroma2.it (G.P.); 5 National Institute for Nuclear Physics (INFN), sez. Roma Tor Vergata, Via della Ricerca Scientifica 1, 00133 Rome, Italy; 6 Department of Technological Innovations and Safety of Plants, Products and Anthropic Settlements (DIT), Italian National Institute for Insurance against Accidents at Work, Inail, Piazzale Giulio Pastore 6, 00144 Rome, Italy; a.bedini@inail.it

**Keywords:** glioblastoma, ^90^Y microbubbles, BED

## Abstract

**Background**: Intra-arterial cerebral infusion (IACI) of radiotherapeutics is a promising treatment for glioblastoma (GBM) recurrence. We investigated the in silico feasibility and safety of Yttrium-90-Poly(vinyl alcohol)-Microbubble (^90^Y-PVA-MB) IACI in patients with recurrent GBM and compared the results with those of external beam radiation therapy (EBRT). **Methods**: Contrast-enhanced T1-weighted magnetic resonance imaging (T1W-MRI) was used to delineate the tumor volumes and CT scans were used to automatically segment the organs at risk in nine patients with recurrent GBM. Volumetric Modulated Arc Therapy (VMAT) treatment plans were generated using a clinical treatment planning system. Assuming the relative intensity of each voxel from the MR-T1W as a valid surrogate for the post-IACI ^90^Y-PVA-MB distribution, a specific ^90^Y dose voxel kernel was obtained through Monte Carlo (MC) simulations and convolved with the MRI, resulting in a ^90^Y-PVA-MB-based dose distribution that was then compared with the VMAT plans. **Results**: The physical dose distribution obtained from the simulation of 1GBq of ^90^Y-PVA-MBs was rescaled to ensure that 95% of the prescribed dose was delivered to 95% or 99% of the target (i.e., A95% and A99%, respectively). The calculated activities were A95% = 269.2 [63.6–2334.1] MBq and A99% = 370.6 [93.8–3315.2] MBq, while the mean doses to the target were 58.2 [58.0–60.0] Gy for VMAT, and 123.1 [106.9–153.9] Gy and 170.1 [145.9–223.8] Gy for A95% and A99%, respectively. Additionally, non-target brain tissue was spared in the ^90^Y-PVA-MB treatment compared to the VMAT approach, with a median [range] of mean doses of 12.5 [12.0–23.0] Gy for VMAT, and 0.6 [0.2–1.0] Gy and 0.9 [0.3–1.5] Gy for the ^90^Y treatments assuming A95% and A99%, respectively. **Conclusions**: ^90^Y-PVA-MB IACI using MR-T1W appears to be feasible and safe, as it enables the delivery of higher doses to tumors and lower doses to non-target volumes compared to the VMAT approach.

## 1. Introduction

Glioblastoma (GBM) is the most common malignant neoplasm in the central nervous system which remains an unresolved clinical problem, with a median life expectancy of 15 months following diagnosis and an overall survival rate of approximately 5% at 5 years [1]. Currently, surgical resection followed by external beam radiation therapy (EBRT) and adjuvant chemotherapy is the standard treatment given to patients with GBM. Unfortunately, in patients with recurrent GBM, re-intervention and second-line chemotherapy often prove to be ineffective [2], and tumor re-irradiation has to be performed in higher doses, which can lead to increased neurotoxicity [3]. Brachytherapy with interstitial implants [4,5,6] and stereotactic radiosurgery [7,8,9,10,11,12,13,14] have been reportedly used as additional methods for delivering high-gradient doses; however, the associated risk of neurotoxicity remains a concern, potentially impacting the overall survival even with the use of these techniques.

Despite years of intensive research, there has been little success in identifying alternative approaches to treat GBM recurrences. The advent of modern endovascular interventional techniques has enabled the injection of therapeutic agents via intra-arterial cerebral infusion (IACI).

The core objective of IACI is to deliver a higher concentration of the therapeutic agent directly to the tumor while minimizing systemic side effects. However, the blood–brain barrier (BBB), which regulates the cellular and molecular exchange between blood vessels and brain parenchyma, is highly selective and blocks the passage of ionized molecules whose molecular weights are more than 180 Da [15]. Most chemotherapeutic agents range in size from 200 to 1200 Da. Therefore, despite recent encouraging results [16], the BBB presents a significant obstacle to the effective IACI of chemotherapeutics [17].

Mannitol remains the most effective method for transient BBB disruption, inducing the temporary shrinkage of endothelial cells through the creation of an osmotic gradient [18]. However, its non-selectivity can lead to increased brain fluid, as well as an influx of molecular compounds, resulting in neurological toxicity, aphasia, and hemiparesis [19]. Other approaches to address BBB disruption include the use of bradykinin, a potent vasodilator [19], and Laser Interstitial Thermal Therapy, which induces hyperthermia [20]. However, there is no solid evidence for the efficacy of these methods.

These challenges limit the sole use of chemotherapeutics for effectively treating GBM recurrences. In this context, the ultrasound-mediated stimulation of narrow-sized (3–4 μm) Poly(vinyl alcohol)-Microbubbles (PVA-MBs) has the potential to selectively open the BBB, allowing for the precise control over its extent for approximately 4–6 h post-stimulation [21,22,23]. It is to be noted here that, in a previous study, we demonstrated that ^90^Y-loaded MBs could be functionalized using a specific endothelial target [24], ideally overexpressed in the GBM (i.e., prostate-specific membrane antigen (PSMA) [25,26].

^90^Y is a β radiation emitter capable of penetrating up to 1.2 cm through soft tissue. This makes it suitable for localized radiation therapy targeting the tumor bed, tumor margins, and surrounding microenvironment, which is critical for treating GBM recurrences. This minimally invasive innovative treatment has potential clinical significance, as it can have a broader impact on the patients’ quality of life.

Using an approach similar to liver ^90^Y radioembolization, a pilot study involving the injection of ^90^Y microspheres in canine brain tumors [24] yielded promising results in terms of the efficacy, with tumor reductions ranging from 49% to 94%.

**Scheme 1:**
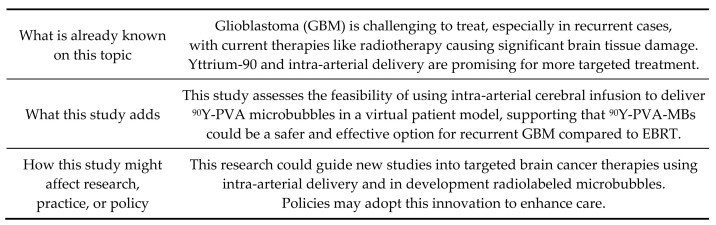
Key Messages.

Similarly, in the current study, we used the method employed by Chen et al. [27] and merged preoperative anatomical MR images with real-time perfusion images to identify the vascular supply to GBM tumors. We then used a custom tool to calculate the absorbed dose distribution from contrast-enhanced MRI images, which served as a surrogate for the ^90^Y-PVA-MB distribution, to investigate the in silico feasibility and safety of the IACI injection of ^90^Y-PVA-MBs to treat patients with recurrent GBM. Our second objective was to compare the ^90^Y-PVA-MB dose distribution with the one calculated using the EBRT approach to assess the equivalent activity required to meet the absorbed dose thresholds for organs at risk (OARs). The key messages are summarized in Scheme 1.

## 2. Materials and Methods

For this study, we assumed that a given administered activity of ^90^Y-PVA-MBs distributes similarly to the medium contrast in the MRI images, akin to the methodology used for liver radioembolization [28], which predicts that a pre-treatment 99mTc-macro-aggregated-albumin SPECT/CT-based map mimics the ^90^Y absorbed dose distribution, facilitating personalized pre-treatment planning.

Patients with recurrent GBM multiforme were included in this in silico proof-of-concept study. Inclusion criteria comprised adult patients (≥18 years) with a confirmed diagnosis of recurrent GBM based on contrast-enhanced MRI, presenting with well-defined pathological lesions consistent with tumor recurrence. All of the patients had previously undergone standard-of-care treatment, including surgery, radiotherapy, and chemotherapy, following established guidelines. Furthermore, all of the patients underwent a three-month follow-up MRI to confirm the recurrence and to rule out treatment-related changes, such as pseudoprogression. Exclusion criteria included patients with brain tumors other than GBM, brain metastases, severe systemic conditions that could compromise short-term survival, active infections, or significant concomitant neurological disorders. Patients with suspected radionecrosis were also excluded, as were those with contraindications to contrast-enhanced imaging or extensive post-treatment alterations that could hinder image interpretation.

### 2.1. MRI and Target Delineation

MRI examinations were performed using a 3T MR scanner (Achieva dStream; Philips Medical System, Best, The Netherlands) with a 32-channel phased-array head coil. All of the MRI examinations included three-dimensional T1-weighted (3DT1-w) and Fluid-Attenuated Inversion Recovery (FLAIR) sequences for the simulation and dose distribution calculations. Additionally, diffusion-weighted imaging (DWI), diffusion kurtosis imaging (DKI), and dynamic contrast-enhanced magnetic resonance imaging (DCE-MRI) were acquired to characterize the diffusivity and permeability of the lesions as a part of the standard protocol for evaluating patients with recurrent GBM, though they were not utilized in the present study.

The 3DT1-w images were acquired using inversion recovery turbo field echo (IR-TFE) with the following parameters: acquisition and reconstruction voxel size of 0.8 × 0.8 × 0.8 mm^3^, repetition time (TR) = 7.9 ms, echo time (TE) = 3.5 ms, flip angle (α) = 8°, and field of view (FOV) = 240 × 201 × 184 mm^3^. The FLAIR images were acquired with the following parameters: acquisition and reconstruction voxel size of 0.75 × 0.99 × 4 mm^3^, TR = 11,000 ms, TE = 125 ms, α = 90°, and FOV = 230 × 180 × 150 mm^3^.

The 3D Slicer Software version 4.6.2 was used to visualize the image sets and delineate the tumor volumes [29]. The volumes of interest (VOIs) and tumor margins were manually delineated on post-contrast T1-w images using a morphological FLAIR image as a guide to the non-enhancing volumes. Visually large vessels and bony components were excluded from the VOIs. A highly experienced interventional neuroradiologist selected the point closest to the lesion where the microcatheter could potentially be positioned using the endovascular approach on the 3DT1-w sequences.

### 2.2. Treatment Planning

The planning computed tomography (CT) image was acquired using a 128-slice Revolution EVO (Lightspeed; General Electric Healthcare, Waukesha, WI, USA). The involved OARs (i.e., brainstem, chiasm, right and left cochlea, globes, lens, and parotid) were automatically segmented using the Limbus version 1.5.0 (Limbus AI Inc., Regina, SK, Canada) on the patient CT images, and subsequently revised and modified by an expert neuroradiologist.

MRI and planning CT images were rigidly fused using MIM Vista software, version 7.1.4 (MIM Software Inc., Beachwood, OH, USA) and the delineated OARs were transferred to T1-w MRI images, while the target (planning target volume, i.e., PTV) was transferred from T1-w MRI to CT images.

Treatment planning calculations were performed for both the ^90^Y-PVA-MBs and EBRT using Volumetric Modulated Arc Therapy (VMAT), as described in the subsections “Dose Calculation with ^90^Y-Labeled PVA-MBs” and “Dose Calculation with VMAT”, respectively.

### 2.3. Dose Calculation with ^90^Y-Labeled PVA-MBs

The dose distribution in tumoral and adjacent non-tumoral tissues was calculated using a dose voxel kernel (DVK) method. The specific ^90^Y kernel was obtained through a custom-designed MC simulation, modeling the particle transport and interaction with tissue-equivalent material, using DOSExyz, which is a well-validated MC code for medical applications [30,31,32,33,34]. For the MC simulation, approximately 5 × 10^8^ primaries were used to simulate the decay events, with a specific energy at a voxel dimension of 0.8 × 0.8 × 0.78 mm^3^. The calculation grid consisted of 53 × 53 × 53 voxels. The simulation characteristics were chosen to limit the uncertainty to less than 1% within 4.8 mm from the central voxel, and were subsequently converted into the average dose to the target voxel per unit of cumulated activity (mGy·MBq^−1^·s^−1^).

The absorbed dose distribution was obtained by assuming that the relative intensity at the voxel levels from the contrast-enhanced MRI T1-w images served as a valid surrogate for the ^90^Y-PVA-MB distribution post-AI. The relative intensity map was convolved with the DVK using a custom MATLAB (R2021b, The MathWorks Inc., Natick, MA, USA) script.

This process is described in Equation (1) below, as follows:

(1) $$\begin{array}{l} D\;\left(x,\;y,\;z\right)=\;\frac{1}{\lambda }\left(A⊗DVK\right)\;\left(x,\;y,\;z\right)\\ \;=\frac{1}{\lambda }\sum_{x^{\prime }}\;\sum_{y^{\prime }}\;\sum_{z^{\prime }}\;A\left(x^{\prime },\;y^{\prime },\;z^{\prime }\right)DVK\left(x-x^{\prime },\;y-y^{\prime },\;z-z^{\prime }\right) \end{array}$$

where *λ* is the decay constant of ^90^Y, *A* represents the 3D activity concentration voxel space, and *DVK* is the computed DVK per disintegration of ^90^Y, which was computed as previously described.

The custom MATLAB tool allowed for the import of the post-contrast T1-w images with tumor segmentations in DICOM format and the ^90^Y DVK obtained from the MC simulation. The absorbed dose distribution was then obtained by convolving the tumor volume with the calculated DVK and applying the appropriate conversion factors based on the physical characteristics of the deposition process. The calculated dose map was aligned to the anatomical post-contrast T1-w acquisition and used to generate the RT dose DICOM file, which was subsequently imported into the MIM software.

### 2.4. Dose Calculation with VMAT

CT images were used to generate VMAT plans with two coplanar arcs using the Pinnacle treatment planning system version 16.4.3 (Philips Medical Systems, Fitchburg, WI, USA). All of the plans were optimized using the Pinnacle auto-planning module to deliver a mean dosage to the PTV of 60 Gy in 30 fractions, while fulfilling the treatment goals adopted in clinical practice [35].

### 2.5. Dose Metrics and Radiobiology

The RT dose DICOM maps generated using ^90^Y-labeled MBs and VMAT were imported into the MIM software to assess the Dose Volume Histogram (DVH) and other dose metrics (e.g., the mean absorbed doses and dose coverage) for the tumor and OARs.

The biological effective dose (*BED*), based on the linear–quadratic model, was calculated for the EBRT using Equation (2), as follows:

(2) $${BED}_{EBRT}=D_{EBRT}\cdot \left[1+\frac{d_{EBRT}}{\alpha /\beta }\right]$$

where *D* is expressed in Gy, *α* and *β* are tissue-specific parameters related to the cell radiosensitivity (expressed in Gy-1 and Gy-2, respectively), while *D_EBRT_* and *d_EBRT_* are the total dose and the dose per fraction of the considered EBRT treatment, respectively.

The *BED* delivered to the target and OARs using ^90^Y-PVA-MBs was obtained using Equation (3), as follows:

(3) $${BED}_{90Y}=D_{90Y}\cdot \left[1+\left(\frac{\lambda }{\lambda +µ}\right)\frac{D_{90Y}}{\alpha /\beta }\right]$$

where *D_90Y_*, expressed in Gy, is the dose administered in a single fraction (N = 1), *µ* is a parameter incorporating the repair of sublethal radiation damage and *µ* = ln(2)/T_1/2,rep_ (expressed as h^−1^) is the repair half-time of sublethal damage, while *λ* is the biologically effective decay constant *λ* = ln(2)/T_1/2,phys_ (expressed as h^−1^). The alpha-to-beta ratio (i.e., *α*/*β*) was set at 10 and 2.5 Gy for tumor and normal tissues [35], respectively, while T_1/2,rep_ was 1.5 and 2.5 h for tumor and normal tissues [36], respectively, and T_1/2,phys_ was 64 h. For more details, refer to [27], which used the same approach.

The activity of the ^90^Y-PVA-MBs’ selective IACI was estimated to ensure the same tumor BED as the VMAT treatments (as a surrogate for efficacy), while the Brain-PTVs’ BED was calculated as a surrogate for the toxicity. Details on the calculation of the BED for the PVA-MBs and VMAT performed at the voxel level are reported in [37].

To account for the dose heterogeneity of the ^90^Y distribution in the PTV, we calculated the ^90^Y activity required to guarantee the coverage of both 95% and 99% of the PTV, which was compared to the VMAT plans with a 95% PTV coverage.

The paired *t*-test comparing the mean doses and BED to the PTV and brain across the developed treatment plans was calculated. A *p*-value < 0.05 indicated statistical significance. The analysis was conducted using RStudio v.1.2.1335 (R Core Team, Vienna, Austria; *t*-test function).

## 3. Results

### 3.1. Virtual GBM Patient Cohort

Nine patients, selected as per the aforementioned inclusion criteria (Section 2), underwent in silico proof-of-concept analysis. The main characteristics of the patients are shown in Table 1. Tumor localizations were present in the left and right parietal–temporal lobes, left and right temporal poles, insula, left temporal–occipital lobe, right frontal–parietal–temporal lobe, basal ganglia, and cerebral and right paratrigonal white matter.

### 3.2. Dose Voxel Kernel

The DVK for ^90^Y using the voxel size of the MRI images was obtained using the MC, assuming the deposition of 1 Bq of ^90^Y, primarily dominated by the beta component up to about 0.72 cm and by the gamma component at longer distances.

### 3.3. Dose Calculation and Comparison with EBRT-VMAT

The optimized VMAT plans allowed for the coverage of the PTV with the prescribed dose (i.e., 60 Gy) while fulfilling the dose constraints of the OARs. All of the optimized plans were verified to be deliverable. To enable further comparison of the DVH and dose metrics, we rescaled the activity to be injected to ensure a coverage of 60 Gy to 95% and 99% of the PTV, termed A95% and A99%, respectively.

Figure S1 (in Supplementary Materials) illustrates the heterogeneity of the absorbed dose distribution calculated using our approach, where the total activity of 1GBq of the ^90^Y-PVA-MBs was rescaled to A95% and A99%.

**Figure 1:**
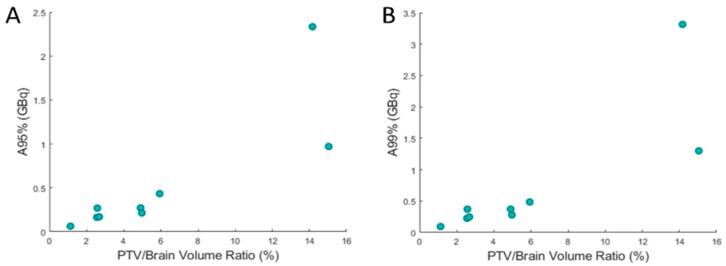
Scatterplot of calculated activities necessary to achieve (**A**) 95% and (**B**) 99% of the prescribed dose to each planned target, i.e., A95% and A99%, respectively.

Figure 1A,B show the prescribed ^90^Y-PVA-MB activities (i.e., A95% and A99%) versus the PTV/normal brain volume ratio, respectively.

The calculated activities’ median values [range] were A95% = 269.2 MBq [63.6–2334.1] and A99% = 370.6 MBq [93.8–3315.2]. In other words, the activity necessary to achieve a minimum dosage covering at least 99% of the PTV was higher than that required for 95% coverage. The difference between A99% and A95% increased with the ratio of the PTV and normal brain volume. The higher the PTV/brain volume ratio, the greater the activity to be injected and the heterogeneity in the absorbed dose distribution.

**Figure 2:**
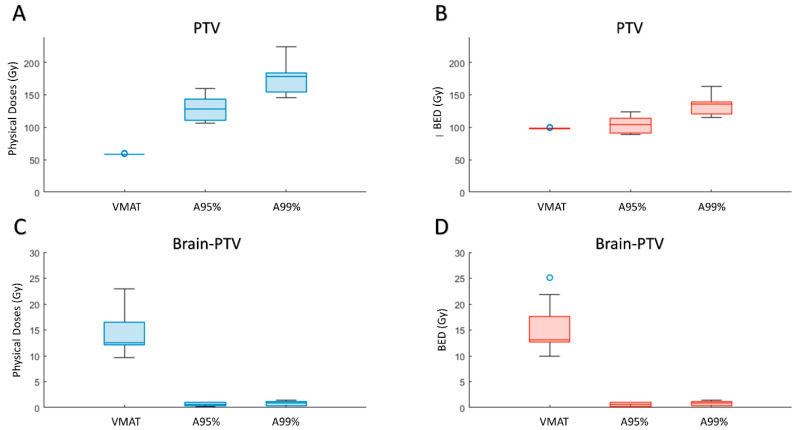
Boxplot of the mean physical doses delivered to the (**A**) PTV and (**C**) Brain-PTV, and the mean BED delivered to the (**B**) PTV and (**D**) Brain-PTV.

The boxplot of the mean physical doses to the PTV and Brain-PTV for the entire patient cohort is presented in Figure 2A,C, respectively, while the corresponding BEDs are shown in Figure 2B,D, respectively. The mean absorbed doses for the PTVs were statistically significantly higher for the 90Y-PVA-MBs than for the VMAT treatments (Figure 2A), while, for the Brain-PTV, they were statistically significantly lower (Figure 2C). The mean BEDs were indistinguishable for the PTV when comparing the 90Y-PVA-MB and VMAT treatments (Figure 2B), confirming their iso-effectiveness, while the values for the Brain-PTV were statistically significantly lower (Figure 2D).

### 3.4. Virtual Cohort Assessment

Table 2 summarizes the mean doses and BED to PTV and Brain-PTV according to the treatment technique and coverage, respectively. Table 3 presents the *p*-values resulting from the *t*-test comparisons of all of the above-reported metrics.

**Figure 3:**
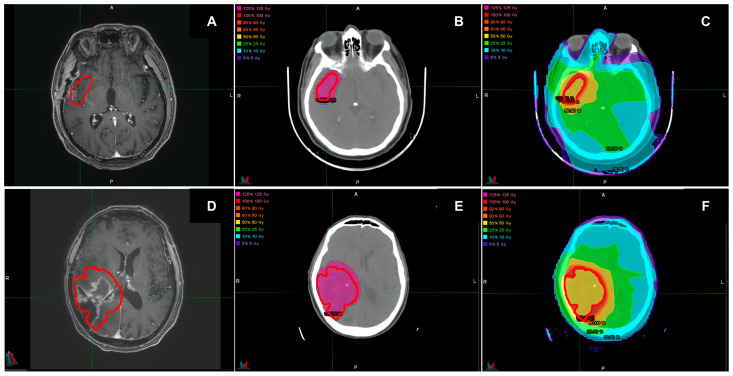
MRI images (**A**,**D**) and BED distribution obtained with ^90^Y-PVA-MBs (**B**,**E**) and VMAT (**C**,**F**) for patient #9 with an intermediate target volume (**A**–**C**) and patient #3 associated with the highest calculated ^90^Y-PVA-MB activity (**D**–**F**). Red lines represent the PTV volumes.

Examples of the ^90^Y-based BED distributions are shown in Figure 3 for a representative patient (Pt#9) with a median target volume (B) and for a patient (Pt#3) characterized by the highest calculated activity (E). The BED distributions of the VMAT approach for these patients are reported in Figure 3C,F.

**Figure 4:**
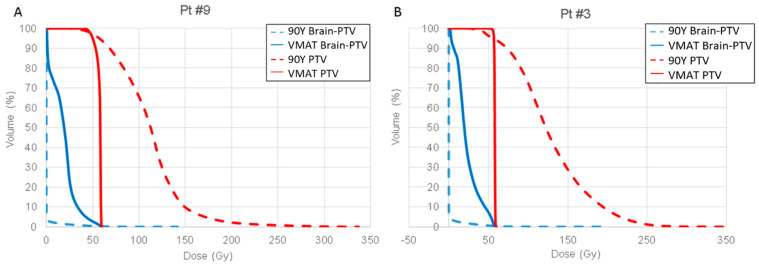
DVHs of the PTV and Brain-PTV for patient #9 with an intermediate target volume (**A**) and patient #3 associated with the highest calculated ^90^Y-PVA-MB activity (**B**). Both of the DVHs were normalized to achieve a 95% coverage of the PTV volume, assuming an injection corresponding to A95% with the ^90^Y-PVA-MBs.

The DVHs of the two patients reported in Figure 3 are shown in Figure 4, highlighting the strong heterogeneity in the dose distribution associated with the ^90^Y-PVA-MB treatment.

Notably, the target coverage achieved using ^90^Y-PVA-MBs depends on the tumor volume and vascularity, whereas VMAT provides homogenous target coverage.

## 4. Discussion

GBM recurrence remains a significant issue due to limited treatment options and poor prognosis [1]. The proof of concept for ^90^Y radioembolization based on the injection of ^90^Y microspheres in canine brain tumors [24] yielded promising results in terms of the efficacy, with tumor reductions ranging from 49% to 94%. However, translating these results to humans may be challenging due to the differences in geometrical and dosimetric characteristics. Rodents, such as mice and rats, are often used as experimental models in dosimetry to assess the absorption and effects of ionizing radiation. Their small size and physiological similarities to humans in certain biological aspects allow researchers to study the dose distribution, tissue damage, and biological responses to varying levels of exposure. However, the key limitations of such animal models relate to the anatomical and physiological differences in the BBB and cerebral vasculature [38,39]. Rodents often exhibit a more homogeneous BBB response to disruption strategies, while humans show inter-patient variability in BBB permeability, regional heterogeneity within the tumor, and an increased risk of neurological adverse effects during BBB modulation procedures.

A potential strategy for improving the understanding of the physiological differences between rodents and humans in the BBB and cerebral vasculature is the adoption of imaging as biomarkers (e.g., dynamic contrast-enhanced MRI or PET) to personalize the BBB modulation and assess the drug delivery heterogeneity within individual patients. Additionally, imaging using radiolabeled PSMA molecules (e.g., ^68^Ga-PSMA), which are overexpressed in the neo-vasculature of the GBM, can enhance the tumor identification and support the selectivity of the proposed approach while reducing the systemic toxicity. To overcome these limitations, future clinical studies should be conducted with these potential improvements in mind.

Traditional systemic chemotherapy, such as temozolomide, achieves cerebrospinal fluid concentrations that represent approximately 20% of plasma levels, leading to suboptimal tumor exposure. In contrast, IACI has been shown to achieve up to 50-fold higher local drug concentrations within the brain parenchyma while minimizing the systemic toxicity [15]. A recent study reported that the intra-arterial delivery of bevacizumab resulted in a 3.6-fold increase in local drug concentration compared to intravenous administration [16].

Localized ^90^Y radiation therapy, enabled by radiolabeled MBs, offers a radiation penetration depth of approximately 1.2 cm, effectively targeting the infiltrative tumor margins while sparing healthy brain tissue. This spatial control is particularly advantageous compared to external beam radiation, which often necessitates broad treatment fields with associated neurotoxicity risks.

We believe that combining systemic chemotherapy with ^90^Y radiation therapy reduces the toxicity compared to the EBRT approach due to the smaller volume of irradiated non-target brain tissue. Another potential advantage of IACI is its minimally invasive nature, which may benefit GBM patients facing recurrence. Owing to the potential for enhancing patients’ quality of life, this approach holds clinical relevance for further research and translation into phase I/II clinical trials. Following the hypothesis of Pasciak AS et al. [24], we used MRI images as a reliable surrogate of the ^90^Y glass microsphere activity distribution map in GBM patients. We investigated the activity of ^90^Y-PVA-MBs to be administered via IACI to achieve the same BED to PTV as the EBRT-VMAT approach. Thus, we conducted an in silico study using a virtual cohort of nine patients to calculate the doses and BED to Brain-PTV volumes, establishing brain toxicity considering radiobiological models for ^90^Y.

Compared to the results reported in [24], the doses calculated for our virtual cohort differed significantly from those calculated for canine models due to the relatively large extent of the disease in the analyzed cases of virtual patients with recurrent GBM. Notably, these differences in the relative geometry could have led to increased doses of normal tissues in animal models when compared to GBM recurrent patients.

Considering the target volume, VMAT treatment guarantees good and homogenous distribution, while the ^90^Y-MB approach shows a heterogeneous dose distribution, as the mean and maximum penetration of the beta particles emitted by ^90^Y are much smaller than the lateral penumbra of the high-energy photon beams used for VMAT delivery.

The dose distribution of ^90^Y-PVA-MBs benefits from passive targeting and has the ability to recognize tissues that may indirectly hit the GBM recurrent cells. Furthermore, the sparing of the Brain-PTV in the ^90^Y-PVA-MB treatment volumes is ensured by the fall-off of the dose profile, calculated using the DVK for the voxel size of the MRI images and the distribution of the contrast agent in the MRI.

Regarding the type of emitter investigated in this study, beta particles have a greater range than alpha particles, potentially delivering higher physical and biological doses while reaching tumor cells located up to 2 cm away, which can be appropriate for treating recurrent GBM tumors.

The use of biocompatible polymer-shelled MBs ensures a very low polymer mass of PVA, along with a good loading capacity of ^90^Y. The small dimensions of the MBs prevent them from lodging in the capillary beds of the tumor, thus avoiding GBM vessel occlusion and its related risk of cerebral ischemia, while further limiting the potential for neoangiogenesis induced by tumor vessel embolization. The ability to target tumor vessels allows the proposed MB radiotherapeutic carrier to adhere to the GBM vessel wall, functioning as a permanent implant owing to the lengthy half-life of ^90^Y.

The primary limitation of this study is that it is an in silico feasibility study conducted to establish the activity linked to the new PVA-MB prototypes to be tested on animals and further optimized for human use to prevent potential brain ischemia. However, the effects of hot spots in absorbed doses and BEDs within the PTV should be further investigated in preclinical studies before translating them into human studies.

In the future, the advantages highlighted in using MBs as carriers of ^90^Y could be combined with the well-known multimodal imaging capabilities of MBs, leading to a potentially invaluable theragnostic antitumor strategy.

## 5. Conclusions

Our results suggest that the lower mean dose to the brain compared with EBRT might be a less toxic and more effective treatment compared to the higher mean and maximal dose delivered by beta emitters.

Overall, considering the poor prognosis of patients with recurrent GBM and the potential toxicity associated with conventional radiotherapeutic approaches, the proposed ^90^Y-PVA-MB IACI treatment appears to be capable of increasing the mean target dose while sparing OARs. These advantages may be particularly beneficial in cases of large GBM recurrences, where radio-resistant tumor cells require an increased mean dose while sparing the non-target tissues.

## Figures and Tables

**Table 1:** Baseline characteristics of the GBM patient cohort used for the in silico study.

Characteristic	Values
Sex (M/F)	7/2
Age (mean; range)	63.7; 43–77 years
Tumor volumes (median; range)	53.6; 16.5–212.1 mL

**Table 2:** Comparison of the mean doses to the PTV and Brain-PTV volumes for the plans according to the treatment technique and target coverage (#, patient identification number).

Pt #	PTV Mean Dose			Brain-PTV Mean Dose		
	VMAT (Gy)	A_95%_ ^90^Y-PVA-MBs (Gy)	A_99%_ ^90^Y-PVA-MBs (Gy)	VMAT (Gy)	A_95%_ ^90^Y-PVA-MBs (Gy)	A_99%_ ^90^Y-PVA-MBs (Gy)
1	60.0	159.4	178.1	9.9	1.0	1.1
2	60.0	133.6	178.9	15.5	0.2	0.3
3	60.0	128.6	182.7	23.6	1.0	1.5
4	60.0	117.6	161.3	11.9	0.6	0.8
5	60.0	153.9	223.8	12.9	0.6	0.9
6	60.0	139.5	186.5	12.2	1.0	1.4
7	60.0	106.9	146.9	12.5	0.3	0.4
8	60.0	112.1	145.9	12.9	0.7	0.9
9	60.0	107.0	157.0	20.7	0.2	0.3

**Table 3:** The *p*-values resulting from the *t*-test of the comparison of the mean dose and BED values for each plan and prescription approach shown in Figure 2 and partially reported in Table 2.

Compared Variables	*p*-Value
Mean dose to PTV obtained using VMAT vs. A_95%_ ^90^Y-PVA-MBs	5.4 × 10^−6^
Mean dose to PTV obtained using VMAT vs. A_99%_ ^90^Y-PVA-MBs	6.4× 10^−7^
Mean dose to Brain-PTV obtained using VMAT vs. A_95%_ ^90^Y-PVA-MBs	1.4 × 10^−5^
Mean dose to Brain-PTV obtained using VMAT vs. A_99%_ ^90^Y-PVA-MBs	1.5 × 10^−5^
BED to PTV obtained using VMAT vs. A_95%_ ^90^Y-PVA-MBs	8.0 × 10^−2^
BED to PTV obtained using VMAT vs. A_99%_ ^90^Y-PVA-MBs	5.9 × 10^−3^
BED to Brain-PTV obtained using VMAT vs. A_95%_ ^90^Y-PVA-MBs	2.2 × 10^−5^
BED to Brain-PTV obtained using VMAT vs. A_99%_ ^90^Y-PVA-MBs	2.3 × 10^−5^

## Data Availability

The original contributions presented in this study are included in the article/Supplementary Materials. Further inquiries can be directed to the corresponding author.

## Supplementary Materials

The following supporting information can be downloaded at: https://www.mdpi.com/article/10.3390/pharmaceutics17030345/s1, Figure S1. Absorbed dose distribution calculated assuming and administration of 1GBq or rescaling the injected activity to cover the 95% or 99% of PTV. In this example patient, the calculated A95% and A99% were 2.334 and 3.315GBq, respectively.
