# Supplementary material for: Intra-Arterial Super-Selective Delivery of Yttrium-90 for the Treatment of Recurrent Glioblastoma: In Silico Proof of Concept with Feasibility and Safety Analysis
Source: Pharmaceutics. 2025 Mar 7;17(3):345. doi: 10.3390/pharmaceutics17030345 (PMC11945926; doi:10.3390/pharmaceutics17030345)
Supplement: Supplementary file 1 [file pharmaceutics-17-00345-s001.zip › pharmaceutics-3447247-supplementary.pdf]

Intra-Arterial Super-Selective Delivery of Yttrium-90 for the Treatment of Recurrent Glioblastoma: In Silico Proof of Concept with Feasibility and Safety Analysis

Giulia Paolani [1,†], Silvia Minosse [2,†], Silvia Strolin [1], Miriam Santoro [1], Noemi Pucci [3], Francesca Di Giuliano [3], Francesco Garaci [3], Letizia Oddo [4], Yosra Toumia [4,5], Eugenia Guida [3], Francesco Riccitelli [4], Giulia Perilli [4], Alessandra Vitaliti [4], Angelico Bedini [6], Susanna Dolci [3], Gaio Paradossi [4,5], Fabio Domenici [4,5,*], Valerio Da Ros [3,‡] and Lidia Strigari [1,‡]

[1] Department of Medical Physics, IRCCS Azienda Ospedaliero-Universitaria di Bologna, Via Massarenti 9, 40138 Bologna, Italy; giulia.paolani@ausl.re.it (G.P.); silvia.strolin@aosp.bo.it (S.S.); miriam.santoro@aosp.bo.it (M.S.); lidia.strigari@aosp.bo.it (L.S.)

[2] U.O.C Diagnostic Imaging, Department of Integrated Care Processes, Fondazione PTV Policlinico "Tor Vergata", University of Rome "Tor Vergata", Viale Oxford 81, 00133 Rome, Italy;
silvia.minosse2@gmail.com

[3] Department of Biomedicine and Prevention, University of Rome "Tor Vergata", Viale Oxford 81, 00133 Rome, Italy; noemi.pucci@students.uniroma2.eu (N.P.); francesca.di.giuliano@uniroma2.it (F.D.G.);
garaci@gmail.com (F.G.); eugenia.guida@uniroma2.it (E.G.); dolci@uniroma2.it (S.D.); darosvalerio@gmail.com (V.D.R.)

[4] Department of Chemical Science and Technologies, University of Rome "Tor Vergata", Via della Ricerca Scientifica 1, 00133 Rome, Italy; letizia.oddo@gmail.com (L.O.); yosra.toumia@roma2.infn.it (Y.T.); fra.riccitelli@gmail.com (F.R.); giulia.perilli@uniroma2.it (G.P.); alessandra.vitaliti@uniroma2.it (A.V.); paradossi@stc.uniroma2.it (G.P.)

[5] National Institute for Nuclear Physics (INFN), sez. Roma Tor Vergata, Via della Ricerca Scientifica 1, 00133, Rome, Italy

[6] Department of Technological Innovations and Safety of Plants, Products and Anthropic Settlements (DIT), Italian National Institute for Insurance against Accidents at Work, Inail, Piazzale Giulio Pastore 6, 00144 Rome, Italy; a.bedini@inail.it

* Correspondence: fabio.domenici@uniroma2.it

† These authors contributed equally to this work.

‡ These authors contributed equally to this work.

Supplementary Material

Figure S1 has been obtained according to the method reported in "Materials and Methods" section, paragraph 2.5 (Dose metrics and radiobiology). These data are cited in the "Results" section, paragraph 3.3 (Dose Calculation and comparison with EBRT-VMAT).

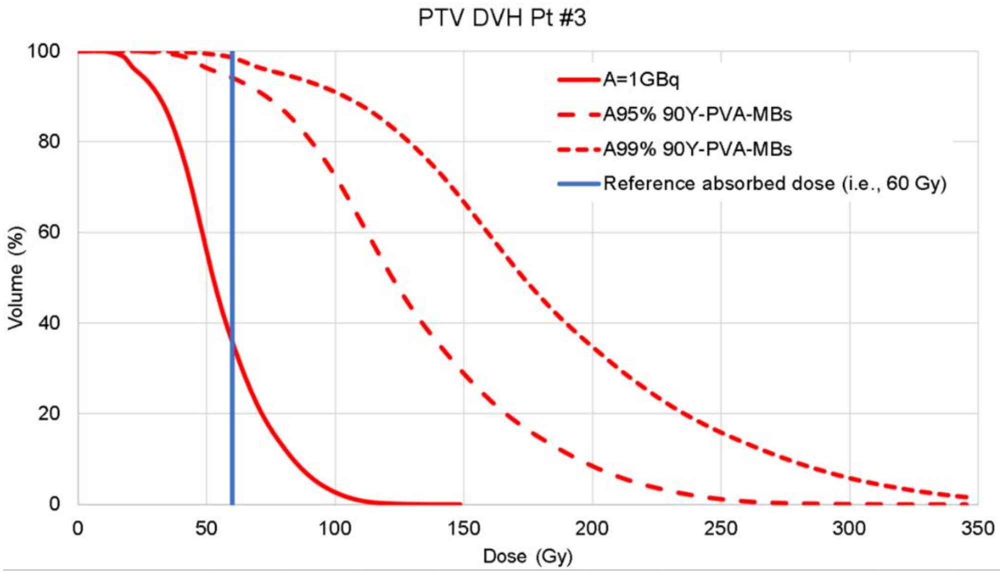

Figure S1. Absorbed dose distribution calculated assuming an administration of 1GBq or rescaling the injected activity to cover the 95% or 99% of PTV. In this example patient, the calculated A95% and A99% were 2.334 and 3.315GBq, respectively.
